# Metastasis-directed therapy: a new standard for oligorecurrent prostate cancer?

**DOI:** 10.18632/oncotarget.26152

**Published:** 2018-09-28

**Authors:** Thomas Zilli, Piet Ost

**Affiliations:** Department of Radiation Oncology, Hôpitaux Universitaires de Genève, Geneva, Switzerland

**Keywords:** prostate cancer, oligometastasis, SBRT, radiotherapy

The STOMP trial is a multicenter, randomized, phase II trial [1] that included 62 patients with oligorecurrent, hormone-sensitive, prostate cancer, defined as up to 3 asymptomatic metastases detected on choline positron emission tomography-computed tomography (PET-CT). Patients were randomized (1:1) to surveillance or metastasis-directed therapy (MDT) with surgery or stereotactic body radiotherapy (SBRT) of all visible lesions. Fifty-five percent of the patients presented a nodal recurrence, mainly located in the pelvis, while the remaining patients mostly had bone metastases. With a median follow-up of 3 years, the MDT group experienced a longer androgen-deprivation therapy (ADT)-free survival compared to the surveillance group (21 vs. 12 months, HR 0.60, 80% CI, 0.40 to 0.90). Biochemical relapse free-survival was also improved with MDT (HR 0.52, 80% CI, 0.36 to 0.76). MDT was well tolerated, with no severe treatment-related toxicity and no detrimental impact on health-related quality of life (HRQoL).

Despite some inherent limitations such as the small patient cohort and the use of a treatment-related primary endpoint, the STOMP trial represents the first randomized study exploring the role of MDT compared to standard of care in oligorecurrent patients. Of note, preliminary results of the phase II randomized Baltimore ORIOLE trial (ClinicalTrials.gov: NCT02680587), comparing observation versus SBRT in oligometastatic hormone-sensitive prostate cancer, are comparable to those observed in the STOMP trial, validating the safety and efficacy of SBRT in this setting.

It is noteworthy that the optimal treatment of oligorecurrent patients remains a challenging and open question, based mainly on, even if promising, retrospective and heterogeneous data. The possibility to treat a metastatic disease with a potentially curative intent collects an increasing interest, with expert panels considering MDT a valid treatment option for these patients [2]. Of note, in a large retrospective, multi-institutional, pooled cohort of nodal oligorecurrent patients Steuber *et al.* observed a significant improvement in cancer specific survival in patients receiving MDT over standard of care (continuous or intermittent ADT) [3]. Some points merit further discussion.

First, although oligorecurrent prostate cancer with exclusive nodal involvement is a common clinical situation [4] and the STOMP and the ORIOLE trials mainly provide evidence on the role of focal SBRT, questions arise on the optimal treatment strategy to adopt in these patients. Treatment strategies using whole-pelvic radiotherapy (WPRT) with elective nodal irradiation and boost to the positive nodes [5], or salvage lymph node dissection (sLND) with or without adjuvant WPRT [6] may be valid therapeutic alternatives probably associated with better progression-free survival rates, although an improvement on the long-term outcome as compared to repeated SBRT has not been demonstrated yet. Moreover, combinations of MDT with standard androgen ablation and temporary systemic therapies such as second line ADT may constitute a strategy to further optimize long-term results of these treatments.

Second, improvements in the diagnostic procedures such as use of ^68^Ga prostate-specific membrane antigen (PSMA) PET-CT [7] will surely help in the next future to better select patients candidates for MDT, by identifying with an improved accuracy recurrent disease and by differentiating low-burden versus polymetastatic disease. Results of the STOMP trial and previous studies using choline PET tracers as well as the role of focal versus more extensive treatment strategies should be therefore interpreted by taking into account these differences in restaging techniques.

Lastly, implementation in trials of translational analysis of leading-edge laboratory measuring circulating tumor cells, circulating tumor DNA, and circulating T-cell receptor repertoires will surely help to better characterize the oligomestatic status by identifying patients with potentially curable metastatic disease and evaluating the effects of MDT on disease response [8].

The recently opened Belgian-Swiss PEACE V STORM trial (ClinicalTrials.gov: NCT03569241), a randomized phase II trial for the Salvage Treatment of OligoRecurrent nodal prostate cancer Metastases, will surely help to answer some of these open questions. By randomizing patients between MDT (SBRT or sLND) versus MDT combined with WPRT (adjuvant WPRT after sLND or WPRT with boost to the positive nodes), and implementing modern imaging techniques and liquid biopsies, the study aims at finding the better treatment strategy in terms of extrapelvic metastasis-free survival as primary endpoint for this subset of patients.

In the context of an individualized multidisciplinary discussion, all patients presenting an oligometastatic recurrence should be considered for MDT in order to reduce disease-burden, postpone disease progression and delay the use of long-term ADT. Enrollment in prospective trials exploring the role of MDT or in the joint European Organization for the Research and Treatment of Cancer (EORTC) and European Society for Radiotherapy and Oncology (ESTRO) OligoCare observational basket study should be encouraged.
